# Supplementary material for: Influenza A Virus Infection Reactivates Human Endogenous Retroviruses Associated with Modulation of Antiviral Immunity
Source: Viruses. 2022 Jul 21;14(7):1591. doi: 10.3390/v14071591 (PMC9320126; doi:10.3390/v14071591)
Supplement: Supplementary file 1 [file viruses-14-01591-s001.zip › MotifAnalysis.zip!/MotifOutput_SC35M/homerResults/motif4.similar.html]

motif4

## Information for motif4

C
G
T
A
A
G
T
C
A
G
T
C
C
T
G
A
C
G
T
A
A
C
G
T
A
G
T
C
C
T
A
G
C
T
A
G
A
G
T
C
  
Reverse Opposite:  

A
C
T
G
A
G
T
C
A
G
T
C
T
C
A
G
C
G
T
A
A
C
G
T
A
G
C
T
A
C
T
G
A
C
T
G
A
C
G
T
  

|  |  |
| --- | --- |
| p-value: | 1e-16 |
| log p-value: | -3.786e+01 |
| Information Content per bp: | 1.894 |
| Number of Target Sequences with motif | 10.0 |
| Percentage of Target Sequences with motif | 24.39% |
| Number of Background Sequences with motif | 134.8 |
| Percentage of Background Sequences with motif | 0.29% |
| Average Position of motif in Targets | 109.1 +/- 40.7bp |
| Average Position of motif in Background | 95.5 +/- 80.8bp |
| Strand Bias (log2 ratio + to - strand density) | 10.0 |
| Multiplicity (# of sites on avg that occur together) | 1.60 |
| Motif File: | file (matrix) reverse opposite |

### Similar de novo motifs found

|  |  |  |  |  |  |  |  |
| --- | --- | --- | --- | --- | --- | --- | --- |
| Rank | Match Score | Redundant Motif | P-value | log P-value | % of Targets | % of Background | Motif file |
| 1 | 0.644 | A G T C C G T A C T G A A C G T A G T C C T A G C G T A A G T C C G T A A G T C | 1e-14 | -33.413032 | 14.63% | 0.03% | motif file (matrix) |
